# Association of Growth-Associated Protein 43 with White Matter Alterations in Patients with Cognitive Decline

**DOI:** 10.21203/rs.3.rs-7004572/v1

**Published:** 2025-08-12

**Authors:** Rasa Zafari, Amirhossein Kamroo, Fardin Nabizadeh

**Affiliations:** Tehran University of Medical Sciences; Tehran University of Medical Sciences; Iran University of Medical Sciences

**Keywords:** Alzheimer’s disease, Mild cognitive impairment, Neurodegeneration, Growth-associated protein 43, Diffusion tensor imaging, Cognitive decline

## Abstract

**Background:**

Alzheimer’s disease (AD) is the most common cause of dementia, characterized by a considerable decline in memory. The aggregation of amyloid-beta (Aβ) plaques and tau tangles is the primary pathology of AD. Recently, growth-associated protein 43 (GAP-43) has been suggested as a reliable biomarker in the early diagnosis of patients with AD continuum.

**Objectives:**

In this study, we aimed to observe the association of white matter (WM) features detected by diffusion tensor imaging (DTI) with the cerebrospinal fluid (CSF) level of GAP-43 in patients with cognitive impairment.

**Methods:**

Information from 132 participants from different ATN groups, including 62 with A−/TN−, 16 with A+/TN−, 30 with A−/TN+, and 24 with A+/TN + pathology were enrolled from the Alzheimer’s Disease Neuroimaging Initiative (ADNI) database. We observed the association of CSF GAP-43 with DTI findings among patients with AD spectrum by using a linear regression model adjusted for age, sex, period of education, and APOE ε4 status.

**Results:**

Our findings suggested a significant association for CSF GAP-43 concentration with WM features in the inferior cerebellar peduncle in the A−/TN− group as well as WM in the cerebral peduncle, anterior corona radiata, and the left sagittal stratum of patients with A+/TN− pathology. In addition, a significant relation was reported between DTI findings in the cingulum cingulate, fornix, body, and splenium of the corpus callosum of patients with A−/TN + with CSF GAP-43 concentration. A similar significant association was shown in the posterior limb of the internal capsule of the A+/TN + group. Moreover, a significant association was found between CSF level of GAP-43 and the performance of A+/TN + and A+/TN−groups in cognitive tests.

**Conclusions:**

Our study observed a significant association between CSF GAP-43 concentration and WM microstructural findings in different brain tracts of patients with various ATN groups, suggesting GAP-43 as a reliable and accurate biomarker in the early detection of patients with cognitive decline. Further longitudinal investigations with other imaging methods can provide more evidence on the role of GAP-43 in the detection of brain damage among patients with AD spectrum.

## Introduction

Alzheimer’s disease (AD) is considered as the most prevalent type of dementia, accounting for about 60–80% of the total dementia cases (1). An approximate number of 50 million cases experience AD during their lifetime. Also, worldwide mortality due to AD is approximately around 1.6 million cases (2). AD is marked as a deterioration in memorization followed by other intellectual deficiencies in speech, time-space comprehension, functionary capabilities, and manner changes, altogether resulting in the gradual deprivation of self-agency (3). In addition, mild cognitive impairment (MCI) is mainly defined as the indicator stage prior to dementia, outlining the transitional stage between the normally functioning intellectuality to the critical stage of dementia (4).

Two main pathologies were considered for AD: 1) intra-cellular aggregation of unnatural highly-phosphorylated proteins of tau. Tau protein complexes further accelerate the development of neurofibrillary tangles (NFTs) through the brain cortex and the associated gray matter of the brain, 2) Extracellular masses of amyloid-beta (Aβ) fibrillary peptides in the structure of plaques (3). In line with the histopathology of AD, AD’s biosignatures are divided into three biform classifications: amyloid-β (A), tau (T), and neurodegeneration (N) (5). In relation to the medical procedure, the ATN classification approach is the backbone for diagnosis and varying treatments of patients with AD continuum (6). It is shown that the status of amyloid-β as “A” can be detected through CSF Aβ or amyloid PET, the status of tau as “T” can be assessed by observing CSF p-tau or tau PET, and the status of neurodegeneration as “N” can be determined by observing atrophies on structural MRI, FDG PET, or CSF total tau concentration (7, 8).

Recent studies have suggested new biomarkers rather than tau and Aβ such as growth-associated protein 43 (GAP-43) as involved molecules in patients with cognitive decline (9). GAP-43 encoding is aberrantly enhanced in the course of neural circuit formation and advancement, specifically in the related cortex and hippocampus section of the full-grown brain (10, 11, 12). Besides, GAP-43 is engaged in the processes of neuronal synaptic plasticity, memorization capabilities, and learning (13, 14, 15, 16). Significantly, the posthumous autopsy of brain dissections stated a decrease of GAP-43 expression through the frontal area and some local increases of GAP-43 expression in the hippocampus region of patients with early AD (17, 18). Moreover, the increased concentration of GAP-43 in the cerebrospinal fluid (CSF) is suggested as a reliable biomarker in the diagnosis of patients with AD continuum (9, 19). Evaluating the potential association between GAP-43 and imaging findings in patients with cognitive decline can provide more evidence on the role of GAP-43 in the early diagnosis of these patients.

Magnetic resonance imaging techniques are widely utilized for neuroscience investigations. Diffusion tensor imaging (DTI) is an imaging method which detects microstructural changes in the white matter (WM) of brain tissue through assessing the movements of water molecules (20). Mean diffusivity (MD) is a DTI value that evaluates the overall level of diffusion in every direction, while fractal anisotropy (FA) specifically quantifies how diffusion varies with direction (21, 22). In addition, Radial diffusivity (RD) measures diffusion perpendicular to the axon, while axial diffusivity (AD) assesses diffusion along the axon (23). It is shown that DTI can reflect neurodegeneration of different brain regions in the early stages of dementia (24).

In this study, we aimed to evaluate the potential association between findings in WM tracts and the CSF level of GAP-43 in patients with AD spectrum to observe the efficacy of GAP-43 as a reliable biomarker in the early detection of patients with cognitive impairment.

## Methods

The data used in this study were obtained from the Alzheimer’s Disease Neuroimaging Initiative (ADNI) database (https://adni.loni.usc.edu/). Launched in 2003 as a collaboration between the public and private sectors, ADNI is led by Principal Investigator Michael W. Weiner, MD. The primary objective of ADNI is to evaluate the effectiveness of repeated magnetic resonance imaging (MRI), positron emission tomography (PET), other biological markers, as well as clinical and neurophysiological evaluations in tracking the progression of MCI and AD.

### Participants

Data on participants, including demographic details, post-processed DTI, and the mean CSF concentration of GAP-43, were collected from baseline visits. This cross-sectional study involved 62 participants with A−/TN− pathology, 16 with A+/TN−, 30 with A−/TN+, and 24 with A+/TN + pathologies. Participants were included if baseline data, such as the CSF level of GAP-43 and post-processed DTI, were available. In line with the 2018 NIA-AA “research framework” for diagnosing AD (7), participants in the ADNI project were grouped based on their biomarker profiles using the A/T/N classification scheme (25). The A/T/N scheme includes three biomarker categories: “A” for aggregated Aβ, “T” for aggregated tau, and “N” for neurodegeneration. Each biomarker group was classified as either negative (−) or positive (+) based on whether the biomarkers were in normal or abnormal ranges. In this study, participants were classified as “A+” if their CSF Aβ1–42 level was below 976.6 pg/ml, “T+” if their P-tau181 level exceeded 21.8 pg/ml, and “N+” if their T-tau level was above 245 pg/ml. To simplify comparisons, the aggregated tau (T) and neurodegeneration (N) groups were combined. Thus, TN negative (TN−) was defined as having both tau and neurodegeneration biomarkers within the normal range (T − and N−, specifically P-tau181 ≤ 21.8 pg/ml and T-tau ≤ 245 pg/ml). Participants were classified as TN positive (TN+) if either the tau (T) or neurodegeneration (N) biomarkers were abnormal (T + or N+, i.e., P-tau181 > 21.8 pg/ml or T-tau > 245 pg/ml). It is important to note that only a small proportion, 5.4%, of the participants showed discrepancies between the tau (T) and neurodegeneration (N) biomarker categories.

### CSF GAP-43 measurements

The GAP-43 analysis was conducted using an in-house ELISA method at the Clinical Neurochemistry Laboratory at Sahlgrenska University Hospital (Mölndal, Sweden) by a certified laboratory technician who was blinded to the clinical data, as previously described (26). All standards and control samples were tested in duplicate. The intermediate precision of the GAP-43 assay was assessed using two quality control human CSF samples (QC 1 and QC 2), which had an intra-assay coefficient of variation (CV) of 5.5% and 11%, and an intraassay CV of 6.9% and 15.6%, respectively. For this study, the initial GAP-43 measurement was used to define the baseline visit in all analyses.

### Diffusion tensor imaging processing

DTI is an MRI technique used to track the movement of water molecules, allowing for the mapping and visualization of white matter (WM) microstructure in the brain (27). The DTI ROI analysis data was sourced from the ADNI database. The preprocessing of the DTI datasets involved the use of the Extraction Tool (BET) in FSL, which corrected for head motion and removed non-brain tissue from the T1-weighted scans of each subject (28). These T1-weighted scans were then aligned to the Colins27 brain template using FSL’s flirt 38,39. The Colins27 brain was initially processed into a cubic isotropic image of size 220 × 220 × 220 mm^3^, which was subsequently down-sampled to 110 × 110 × 110 mm^3^ to match the resolution of the diffusion-weighted imaging (DWI). A diffusion tensor model was applied to each voxel, which enabled the creation of scalar anisotropy and diffusivity maps from the eigenvalues of the diffusion tensor (λ1, λ2, λ3) (29). These maps provided metrics such as fractional anisotropy (FA), mean diffusivity (MD), radial diffusivity (RD), and axial diffusivity (AxD). Lower FA and higher RD, AxD, and MD values are indicative of white matter degeneration and demyelination. To align the FA image from the Johns Hopkins University (JHU) DTI atlas with each individual subject, an elastic registration method based on shared information was used (30, 31). Nearest-neighbor interpolation was employed to avoid label overlap when applying the transformation to the JHU “Eve” WM atlas labels, ensuring that the regions of interest (ROIs) from the atlas corresponded accurately to the DTI maps (http://cmrm.med.jhmi.edu/cmrm/atlas/human_data/fle/AtlasExplanation2.htm). The average FA and MD values were then computed within each subject’s ROI masks. Tensor-based spatial statistics were used to extract mean FA values within the ROIs and along the skeleton (32). The tract-based spatial statistics (TBSS) method, which is an automated, observer-independent approach, was utilized to assess FA in major white matter tracts across different groups, following the ENIGMA-DTI group protocols (enigma.loni.ucla.edu/wpcontent/uploads/2012/06/ENIGMATBSSprotocol.pdf). All subjects were aligned with the ENIGMA-DTI template in ICBM space, a probabilistic white matter atlas, and standard TBSS procedures were followed to project the individual FA maps onto the skeletonized ENIGMA-DTI template, with ROI extraction for the mean FA along the skeleton (http://enigma.loni.ucla.edu/wpcontent/uploads/2012/06/ENIGMA_ROI_protocol.pdf.).

### Cognitive assessments

To assess the cognitive functioning of participants, the MMSE was used, which measures several cognitive areas including orientation, attention, language, memory, and visual-spatial skills. Furthermore, the Montreal Cognitive Assessment (MoCA) and the Alzheimer’s Disease Assessment Scale-Cognitive Subscale (ADAS-Cog) were also applied to evaluate the level of cognitive decline among the participants. Information regarding the participants’ scores on the MMSE, MoCA, and ADAS-Cog was obtained from the ADNI database.

### ApoE genotyping and CSF GAP-43 measurements

Blood samples were examined for APOE genotyping and CSF GAP-43 levels, and the findings can be accessed via the ADNI database (adni.loni.usc.edu/methods/documents/). Following ADNI protocols, individuals with one or more ε4 alleles are classified as APOE ε4 carriers.

### Statistical analyses

We performed the statistical analysis using SPSS16 software. First, the normality of the variables was assessed using the Kolmogorov-Smirnov and Shapiro-Wilk tests. Variables that were not normally distributed were log-transformed to achieve normality. A one-way ANOVA with Bonferroni correction was then used to compare group differences. To examine the relationship between plasma NT1 levels and demographic and clinical variables, we applied a simple linear regression model, which helped to identify covariates for further analysis. We then conducted multivariable linear regression models to explore the association between plasma NT1 (as the dependent variable) and DTI parameters in each ROI (as independent variables), adjusting for confounders identified earlier. To correct for multiple comparisons and avoid type I errors in the correlation models, we used the Benjamini-Hochberg correction method.

## Results

### Participants characteristics

**Figure 1:**
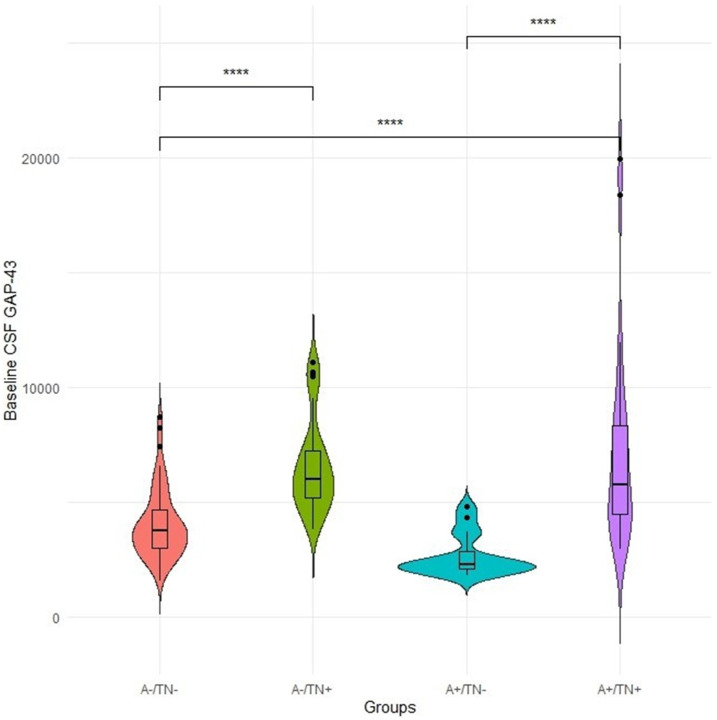
Baseline CSF level of GAP-43 among the ATN groups. CSF: Cerebrospinal fluid, GAP-43: Growth-associated protein 43.

The baseline cohort data of 132 participants was used in this study. The mean age of participants was 71, consisting of 66 females and 66 males. The mean length of education was 16.5 years. Also, participants reflected an average MoCA score of 24.77, an average MMSE of 28.80, a mean ADAS11 score of 6.89, and a mean ADAS13 score of 10.69. Furthermore, it is shown that 36 participants reflect at least one APOE ε4. A significant difference in the CSF level of GAP-43 was observed between four groups [F (3,131) = 20.611, P < 0.001] (Fig. 1). On the other hand, unlike years of education [F (3,131) = 3.170, P = 0.027], no significant differences were found in age among evaluated groups [F (3,131) = 1.375, P = 0.254]. Considering APOE ε4 status, a significant difference was observed between the four groups [F (3,131) = 7.841, P < 0.001]. Unlike MMSE [F (3,131) = 2.628, P = 0.053] and MoCA [F (3,131) = 0.537, P = 0.657], significant differences were reported in the performance of four groups in ADAS11 [F (3,131) = 5.842, P < 0.001] and ADAS13 [F (3,131) = 6.937, P < 0.001]. Table 1 summarizes the demographic information of participants.

### Baseline differences of GAP-43 CSF levels among the ATN groups

As mentioned before, our analysis observed a significant difference in the CSF level of GAP-43 between ATN groups [F (3,131) = 20.611, P < 0.001]. In addition, the post-hoc tests revealed significant differences between most of the introduced ATN groups (P < 0.001) (A−/TN− vs A−/TN+, A−/TN− vs A+/TN+, A+/TN− vs A−/TN+, and A+/TN− vs A+/TN+).

### Association of GAP-43 CSF level and cognitive tests

**Figure 2:**
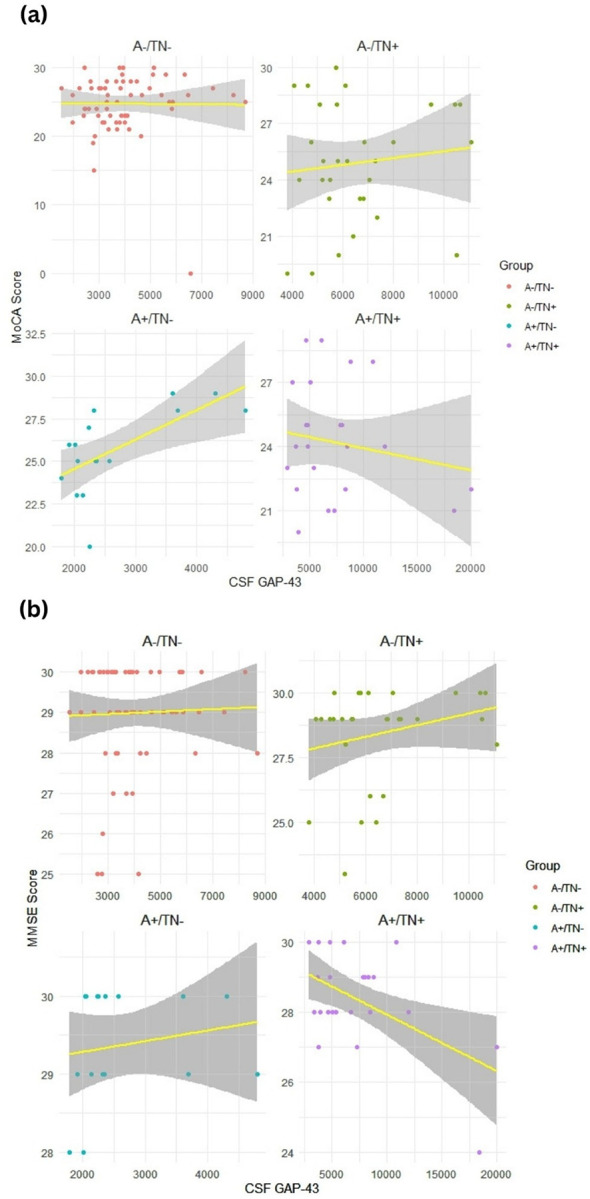
Association of GAP-43 CSF concentration and cognitive performance of participants with different ATN groups. a) Association of GAP-43 and MoCA score. b) Association of GAP-43 and MMSE score. CSF: Cerebrospinal fluid, GAP-43: Growth-associated protein 43. MMSE: Mini-Mental State Examination, MoCA: Montreal Cognitive Assessment

After adjusting our analysis for age, sex, years of education, and APOE ε4 status, we observed a significant correlation between the CSF level of GAP-43 and the performance of participants with A+/TN−pathology in MoCA assessment [β = 0.644, P = 0.007] (Fig. 2a), as well as the performance of participants with A+/TN + pathology in MMSE evaluation [β= − 0.524, P = 0.005] (Fig. 2b).

### Association of GAP-43 CSF level and demographic features

Our linear regression reflected no significant associations between the CSF level of GAP-43 and age (P = 0.927) and the education period of participants (P = 0.120). Also, it is shown that CSF GAP-43 is not significantly associated with the whole brain (P = 0.069) and hippocampal volumes (P = 0.129). However, a significant association between the CSF level of GAP-43 and MMSE score (P = 0.046), as well as the APOE ε4 status (P = 0.008), was reported among participants.

### Association of GAP-43 CSF level and white matter microstructural findings among the ATN groups

**Figure 3:**
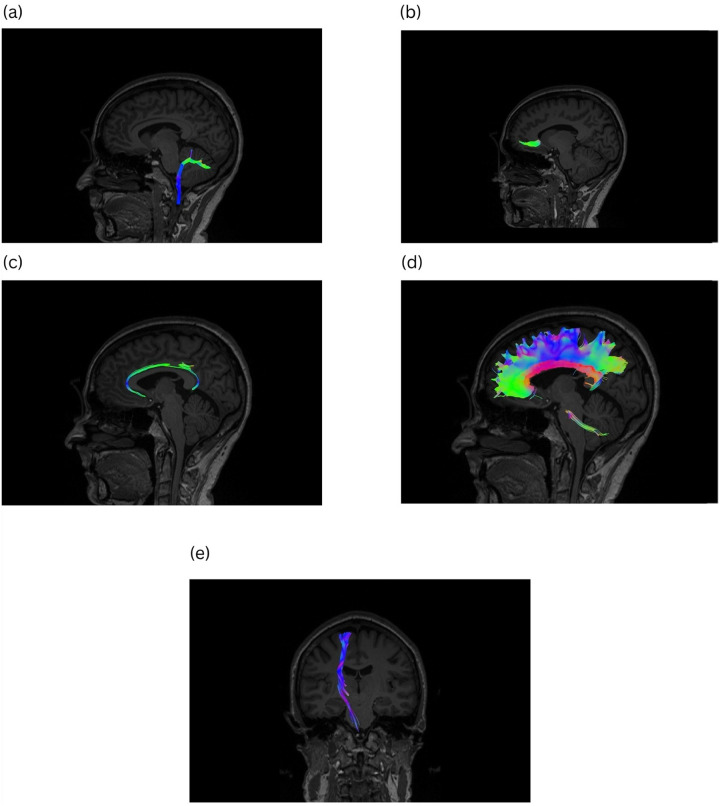
White matter tracts that reflect DTI values associated with CSF GAP43 concentration in patients with cognitive decline (p value < 0.05). a) Inferior cerebellar peduncle b) Anterior corona radiata c) Cingulum cingulate d) Corpus callosum e) Posterior limb of internal capsule. CSF: Cerebrospinal fluid, GAP-43: Growth-associated protein 43, DTI: Diffusion tensor imaging

We conducted a univariate multiple regression model including age, sex, period of education, APOE ε4, and GAP-43 CSF level to assess the potential association between white matter alterations detected by DTI and the CSF concentration of GAP-43. Our analysis revealed a significant positive association between the FA of the left inferior cerebellar peduncle (ICP), right ICP, and bilateral ICP as well as a negative association for the RD of these tracts and the CSF level of GAP-43 among participants with A−/TN− pathology. Moreover, it is shown that the FA of the left ICP, left cerebral peduncle (CP), bilateral CP, left anterior corona radiata (ACP), and left sagittal stratum (SS) is significantly associated with CSF GAP-43 concentration in participants with A+/TN− pathology. This ATN group also reflected a significant relation between the RD of left SS and left cingulum cingulate (CGC) and the level of GAP-43 in the CSF. Furthermore, our analysis observed a significant association for the AxD of bilateral fornix (FX), bilateral body of corpus callosum (BCC), full CC, and bilateral splenium of corpus callosum (SCC) with the CSF concentration of GAP-43 in the A−/TN + group. Regarding the participants in the A+/TN + group, our analysis suggested a significant negative association between the AxD of the right posterior limb of the internal capsule (PLIC), left PLIC, and bilateral PLIC (Table 2). Figure 3 illustrates some of the most important WM tracts which reflect associated DTI values with CSF GAP-43 concentrations (created by MainExploreDTI (33)).

## Discussion

In this study, we assessed the potential association between GAP-43 CSF levels and WM microstructural findings in patients with different ATN groups using the ADNI cohort. We utilized a univariate multiple regression model adjusting for age, sex, years of education, and APOE ε4 status to observe the relation between DTI findings and the CSF concentration of GAP-43. The results of our analysis revealed a significant association between DTI features of WM in the ICP, CP, ACP, SS, CGC, CC, FX, and PLIC and GAP-43 of patients with different ATN groups.

It is demonstrated that GAP-43 is one of the main biomarkers that represents the status of synaptogenesis and neuroplasticity (34). This protein is expressed in various brain regions, such as the hippocampus and entorhinal cortex (19). Recent studies have suggested that the level of GAP-43 is reduced in the frontal cortex and hippocampus of patients with dementia, reflecting the role of this protein and synaptic integrity in cognitive performance (18, 35, 36, 37). Moreover, most recent studies have shown increased CSF levels of GAP-43 in different brain areas of patients with AD continuum, suggesting this protein as a reliable biomarker in the diagnosis and monitoring of patients with cognitive decline (9, 26). In a study by Songhori et al., it is reflected that GAP-43 can be an accurate predictor of synaptic dysfunction and thus impaired cognitive performance in patients with AD spectrum (38). Previous investigations also reported other synaptic proteins which can reflect cognitive decline in patients with early AD. TREM2 is a synaptic protein which is expressed mainly in the hippocampus (39). It is shown that this protein is significantly associated with tau and amyloid β pathologies in participants with A+/TN+ (40). Postsynaptic density protein 95 (PSD-95) is another synaptic protein playing an essential role in the retention of dendritic synapses (41). Recent studies have observed a significant correlation between decreased expressions of PSD-95 and cognitive impairments in patients with AD continuum (42, 43). The results of our study also suggested a significant difference for CSF GAP-43 concentration among different ATN groups, with the highest levels recorded in the A+/TN + group.

Some studies attempted to evaluate the potential correlation between CSF GAP-43 and cognitive function of patients with AD continuum assessed by cognitive tests like MMSE. Sandelius et al. reported a weakly significant association between CSF GAP-43 levels and the performance of participants in the MMSE assessment (26). Another study evaluating cognitive decline using the Functional Activities Questionnaire (FAQ) evaluation demonstrated a significant correlation between the baseline CSF levels of GAP-43 and FAQ scores among patients with cognitive impairment (44). Our study also reported a significant association between GAP-43 CSF concentration and MMSE in the A+/TN + group, as well as with the MoCA score in the A+/TN− group. Furthermore, some studies assessed the association of GAP-43 concentration in the CSF with tau and amyloid β accumulation in the brain tissue. Franzmeier et al., in their study, reported a significant association between the baseline CSF concentration of GAP-43 and Aβ accumulation rate in the brain of patients with AD spectrum. This study suggested that higher CSF level of GAP-43 is associated with faster Aβ accumulation in patients with early AD (45). Also, another study revealed similar results on the significant association between GAP-43 CSF level and AD pathology, including the accumulation of Aβ and neurofibrillary tangles in patients with dementia, particularly in the hippocampus and cortex (26). Our study also suggested a significant difference in the CSF level of GAP-43 among participants with various ATN pathology groups.

Our study reflected significant findings detected by DTI in WM tracts, including ACR, SS, FX, and PLIC, in relation to CSF GAP-43 concentration among various ATN groups. It is shown that ACR plays an essential role in connecting the thalamus and anterior part of CC (46). This tract is involved in cognitive functions and emotion regulation (47, 48, 49). Wang et al. reported a significant difference in the ACR tract between patients with WM lesions-related dementia and healthy controls (50). This study also reflected a significant correlation between the alteration of the ACR tract and the performance of patients with WM lesions in MoCA assessment (50). Additionally, some studies reported a significant reduction in FA levels of ACR in patients with AD (51). Our study also observed a negative association between the FA of the left ACR and the CSF level of GAP-43 in the A+/TN− group. SS is located on crossroads consisting of several major WM tracts, such as the inferior fronto-occipital fascicle (IFOF) and optic radiation (OR) (30, 52, 53, 54). It is demonstrated that SS is involved in language processing (55), and reading and visual recognition (52, 56). Due to the close correlation between SS and thalamocortical radiations, some studies have suggested that microstructural damages in SS can significantly affect executive functions and cognitive performance in patients with early AD (57, 58). Our study also reflected a significant association between the FA and RD of the SS and the CSF concentration of GAP-43 in the A+/TN− group.

CGC is an essential WM bundle that consists of short and long associative fibers connecting different brain regions (59). It is reported that the cingulum is a main part of the default mode network, which is involved in more complex brain activities (60). Several studies have reported that deficits in default mode network can result in impaired cognition in patients with AD continuum (61, 62). Bozzali et al. reflected a significant correlation between the MD of the cingulum and impairments of episodic memory in patients with cognitive deficits (59). This study also suggested that alterations of the cingulum can be a reliable predictor for the progression of cognitive impairment in patients with dementia (59). Our study also demonstrated a significant association between the RD of the left CGC and the CSF level of GAP-43 in the A+/TN− group. PLIC is a part of the internal capsule that connects the thalamus with different brain areas, including sensory cortex, premotor cortex, and visual association cortex (63). In a study by Canu et al., a significant decrease was found in the FA of PLIC, introducing this tract as one of the most damaged WM tracts in patients with AD (64). We also observed a significant association between the AD of this tract and the concentration of GAP-43 in the CSF of participants with A+/TN + pathology.

Our study included some limitations that are worth mentioning. First, this study did not observe the correlation of CSF GAP-43 concentration and WM alterations longitudinally to provide more accurate information about the role of GAP-43 in predicting the progression of dementia in patients with cognitive decline. In addition, this study did not match participants in different ATN groups for age, sex, race, and genetics, increasing the effect of potential confounding factors on our results. Also, we considered patients with T and N pathology as one group which can reduce heterogeneity among the participants. Finally, other data is needed besides ADNI to provide additional information on participants with longer follow-ups and other imaging methods.

In conclusion, our study suggested a significant association between the CSF level of GAP-43 and microstructural WM findings detected by DTI in patients with different ATN groups suggesting CSF concentration of GAP-43 as a reliable predictive of WM damage as well as cognitive decline in patients with AD continuum. Further studies assessing the longitudinal correlation between GAP-43 level and alterations of WM tracts detected by DTI or even more advanced MRI methods such as Diffusion Kurtosis Imaging (DKI) and High-Angular Resolution Diffusion Imaging (HARDI) can shed more light on our knowledge of the role of GAP-43 in predicting the progression of cognitive deficits in patients with AD spectrum.

## Supplementary Material

Supplementary Files

This is a list of supplementary files associated with this preprint. Click to download.

- GraphicalAbstract.tiff

## Figures and Tables

**Table 1:** Demographic characteristics.

	A−/TN−	A+/TN−	A−/TN+	A+/TN+	P value
	(n = 62)	(n = 16)	(n = 30)	(n = 24)	
**Age (years)**	69.80 ± 6.78	70.52 ± 7.06	72.01 ± 6.28	72.59 ± 6.59	0.254
**Sex (F/M)**	31/31	7/9	17/13	11/13	0.819
**Education (years)**	16.74 ± 2.58	17.50 ± 2.22	16.87 ± 2.90	15.17 ± 2.63	0.27
**MMSE**	28.98 ± 1.30	29.38 ± 0.072	28.43 ± 1.89	28.38 ± 1.35	0.053
**MoCA**	24.69 ± 4.43	25.69 ± 2.47	24.90 ± 3.13	24.21 ± 2.67	0.657
**ADAS11**	6.66 ± 3.27	5.69 ± 2.50	6.03 ± 2.76	9.38 ± 4.50	**< 0.001**
**ADAS13**	9.77 ± 4.86	9.75 ± 3.34	9.67 ± 4.27	14.96 ± 17.07	**< 0.001**
**APOE ε4**					**< 0.001**
**Without ε4**	48	11	20	7	
**One ε4**	12	3	10	12	
**Two ε4**	2	2	0	5	
**Hippocampal volume**	6071.26 ± 3126.02	6041.31 ± 3682.77	5679.70 ± 3059.22	5486.00 ± 2661.69	0.852
**GAP-43 CSF concentration (pg/ml)**	4002.31 ± 1488.70	2649.68 ± 917.31	6570.01 ± 2038.38	7230.99 ± 4387.47	**< 0.001**

Values are showed as mean(± SD) or raw numbers of patients

Results of ANOVA analysis between groups noted as p value and adjusted for age, sex, years of education

A: Aβ pathology, TN: Tau neurodegeneration, APOE ε4: Apolipoprotein E ε4 genotype, MMSE: Mini Mental State Examination, MoCA: Montreal Cognitive Assessments, ADAS-Cog: Alzheimer’s Disease Assessment Scale-Cognitive Subscale, CSF: Cerebrospinal fluid, GAP-43: Growth-associated protein 43.

**Table 2:** Results of linear regression analyses of DTI values and CSF GAP-43 concentration among all participants.

	AxD	FA	MD	RD
**A−/TN−**				
Left inferior cerebellar peduncle	0.158	0.339*	− 0.083	− 0.270*
Right Inferior cerebellar peduncle	0.107	0.271*	− 0.099	− 0.255*
Bilateral Inferior cerebellar peduncle	0.131	0.312*	− 0.094	− 0.269*
**A+/TN−**				
Left inferior cerebellar peduncle	− 0.706	− 0.849*	− 0.306	0.177
Left cerebral peduncle	− 0.493	− 0.956**	0.019	0.596
Bilateral cerebral peduncle	− 0.589	− 0.934*	− 0.236	0.363
Left anterior corona radiata	0.013	− 0.532*	0.187	0.282
Left sagittal stratum	− 0.228	− 0.955*	0.314	0.639*
Left cingulum cingulate	− 0.304	− 0.611	0.007	0.294*
**A−/TN+**				
Bilateral Fornix	− 0.408*	− 0.112	− 0.182	− 0.095
Bilateral body of corpus callosum	− 0.398*	0.070	− 0.262	− 0.184
Full corpus callosum	− 0.359*	0.114	− 0.243	− 0.180
Bilateral splenium of corpus callosum	− 0.336*	0.184	− 0.259	− 0.205
**A+/TN+**				
Left posterior limb of internal capsule	− 0.612*	− 0.368	− 0.358	0.014
Right posterior limb of internal capsule	− 0.746*	− 0.555	− 0.421	0.085
**A−/TN−**				
Bilateral posterior limb of internal capsule	− 0.706*	− 0.485	− 0.410	0.054

* p < 0.05

** p < 0.01

Each cell shows the p-value from the linear regression analysis between DTI metric values of white matter brain regions and CSF GAP-43 levels, adjusted for APOE genotype, years of education, MMSE scores, age, and sex.

A: Aβ pathology, TN: Tau neurodegeneration, AD: Axial diffusivity, FA: Fractional anisotropy, MD: Mean diffusivity, RD: Radial diffusivity

## Data Availability

The datasets analyzed during the current study are available upon request with no restriction.
